# Defining Challenge Conditions for Nipah Virus in the Syrian Golden Hamster Model: Effects of Dose, Exposure Route and Viral Strain

**DOI:** 10.3390/v18091030

**Published:** 2026-09-16

**Authors:** Emma Kennedy, Stephen Findlay-Wilson, Francisco J. Salguero, Ines Ruedas-Torres, Susan Fotheringham, Linda Easterbrook, Victoria A. Graham, Sarah Kempster, Neil Almond, Stuart D. Dowall

**Affiliations:** 1 United Kingdom Health Security Agency (UKHSA), Porton Down, Salisbury SP4 0JG, UK; emma.kennedy@ukhsa.gov.uk (E.K.);; 2 Medicines and Healthcare Products Regulatory Agency (MHRA), South Mimms, Potters Bar, Hertfordshire EN6 3QG, UK

**Keywords:** Nipah, preclinical, challenge, routes, hamster

## Abstract

Nipah virus (NiV) is a highly pathogenic zoonotic virus for which no licensed vaccines or therapeutics are currently available, necessitating well-characterised animal models to support countermeasure development. This study aimed to establish an appropriate challenge dose in the Syrian golden hamster model at the UK Health Security Agency (UKHSA), incorporating both intraperitoneal (i.p.) and intranasal (i.n.) routes of infection and two clinically relevant strains, NiV-Malaysia (NiV-M) and NiV-Bangladesh (NiV-B). Due to a different challenge doses and susceptibilities of hamsters to NiV being reported in the literature, there is a prerequisite to define the model parameters upon establishment in a new facility. Dose-ranging studies were performed across multiple independent experiments assessing survival, clinical progression and histopathological outcomes. Challenge via the i.p. route produced consistent and severe disease using an infectious dose of 10^3^ TCID_50_ for both strains. In contrast, animals were more susceptible to infection via i.n. challenge with a dose of 10 TCID_50_ resulting in uniform lethality. For both challenge routes, respiratory and neurological disease manifestations were observed. These distinct disease phenotypes were supported by histopathological findings and viral RNA distribution in affected organs. Differences between strains were evident, with NiV-B showing a tendency towards a broader pulmonary pathology at lower doses after i.n. challenge. Collectively, these findings demonstrate that challenge dose, route of exposure, and viral strain influence disease outcomes in the hamster model.

## 1. Introduction

Nipah virus (NiV), a highly pathogenic zoonotic paramyxovirus of the genus *Henipavirus*, remains a significant global health concern due to its high case fatality rate (CFR), broad host range, and potential for human-to-human transmission [1]. Its initial emergence during the 1998–1999 outbreak in Malaysia and Singapore resulted in over 265 cases of encephalitis and 105 human deaths, giving a CFR of approximately 40% [2]. Recurrent outbreaks in South and Southeast Asia—predominantly associated with the Bangladesh lineage—have demonstrated distinct epidemiological and clinical differences between strains with CFR’s up to 92% in individual outbreaks [3]. The Malaysian strain (NiV-M) is historically linked with encephalitic disease and limited onward transmission, whereas the Bangladesh strain (NiV-B) is associated with increased respiratory involvement, higher mortality, and more efficient human-to-human spread [4], likely due to different routes of transmission and sources of infection. The incorporation of both strains into preclinical models to reflect infection with the different lineages is therefore an important consideration.

The Syrian golden hamster (*Mesocricetus auratus*) has become a widely used and well-characterised small animal model for NiV infection, as it recapitulates key pathological features observed in humans, including respiratory disease, neurological involvement, and systemic vasculitis. Importantly, this model supports infection via multiple routes, with intranasal (i.n.) and intraperitoneal (i.p.) challenges being among the most frequently employed [5,6,7,8]. These routes can model distinct aspects of NiV pathogenesis: intranasal inoculation more closely mimics natural respiratory exposure, often resulting in prominent pulmonary pathology, whereas intraperitoneal challenge promotes rapid systemic dissemination.

Despite the utility of the hamster model, variability in experimental parameters remains a significant limitation for reproducibility and cross-study comparison. The challenge dose, together with route of administration and viral strain, is a key determinant of disease kinetics, tissue tropism, clinical severity and mortality. Consequently, challenge conditions that produce reproducible and measurable endpoints need establishing for the subsequent effective assessment of interventions. This issue is particularly important in the context of medical countermeasure development under regulatory frameworks. Alternative vaccine licensure pathway allows approval of therapeutics based on evidence which likely would include well-controlled animal efficacy studies when human trials are not ethical or feasible, provided the model is sufficiently well-characterised and predictive of human disease [9]. As such, the establishment of a robust, reproducible, and biologically relevant challenge dose is essential to ensure sensitivity in detecting therapeutic benefit and to support translational relevance.

Whilst it is widely accepted that the hamster model resembles the disease manifestations of human NiV infection, there is a noted variation in challenge doses between institutes [5,8,10]. Therefore, the establishment of the model at the UK Health Security Agency (UKHSA) warranted a series of dose-ranging studies to define the experimental parameters.

## 2. Materials and Methods

### 2.1. Ethical Statement

All experimental procedures with animals were undertaken according to the United Kingdom Animals (Scientific Procedures) Act 1986, with studies conducted under the authority of project licences approved by the UK Home Office. The experimental protocols were approved by ethical review of the UK Health Security Agency’s (UKHSA) Animal Welfare and Ethical Review Body (AWERB), approval references PPL P82D9CB4B and PP3877532. Prior to the start of studies, humane clinical endpoints were identified: 20% weight loss compared to baseline; inactivity/immobility; neurological signs; or on the advice of severe disease from the Named Animal Care and Welfare Officer (NACWO).

### 2.2. Animals

Golden Syrian hamsters (*Mesocricetus auratus*) were obtained from a UK Home Office-accredited supplier (Envigo, Bicester, UK). Adult animals ranging from 5–11 weeks were used for most studies, the exception being six animals that were 18–19 weeks old, which were used due to limitations in animal availability, allocated to Study A for intraperitoneal delivery and split between groups. The initial study (Study A for both the intraperitoneal and intranasal challenge routes) used female animals only, whereas subsequent studies (Studies B–F) used equal allocations of male and female animals for each group. Animals were randomly allocated to groups during the process of identification chipping by staff in the animal facility who were blinded to the study design. Hamsters were housed in groups of *n* = 2–3 per cage. Food and water were available ad libitum, with environmental enrichment provided throughout the study. During challenge procedures, animals were anaesthetised with isoflurane and monitored until a full recovery was observed.

### 2.3. Virus

Nipah virus strains (Malaysian and Bangladesh) were kindly provided by the Special Pathogens branch of the Centers for Disease Control and Prevention, Atlanta, USA. The virus was propagated and titrated by median tissue culture infectious dose (TCID_50_) on VeroE6 cells (European Collection of Cell Cultures, Salisbury, UK) grown using Dulbecco’s minimal essential medium (DMEM; Gibco, Paisley, UK) supplemented with 2% foetal bovine serum (Gibco, Paisley, UK) at 37 °C. All culturing and titration assays were undertaken in a class III biological safety cabinet line in the containment level (CL) 4 laboratory at UKHSA, Porton Down. Stocks were sterility-tested and confirmed negative of mycoplasma contamination. Genome sequencing confirmed consensus sequences aligned to NiV-M and NiV-B (Genbank accession numbers AF212302 and AY988601, respectively).

### 2.4. Study Design

#### 2.4.1. Intraperitoneal Challenge

**Figure 1:**
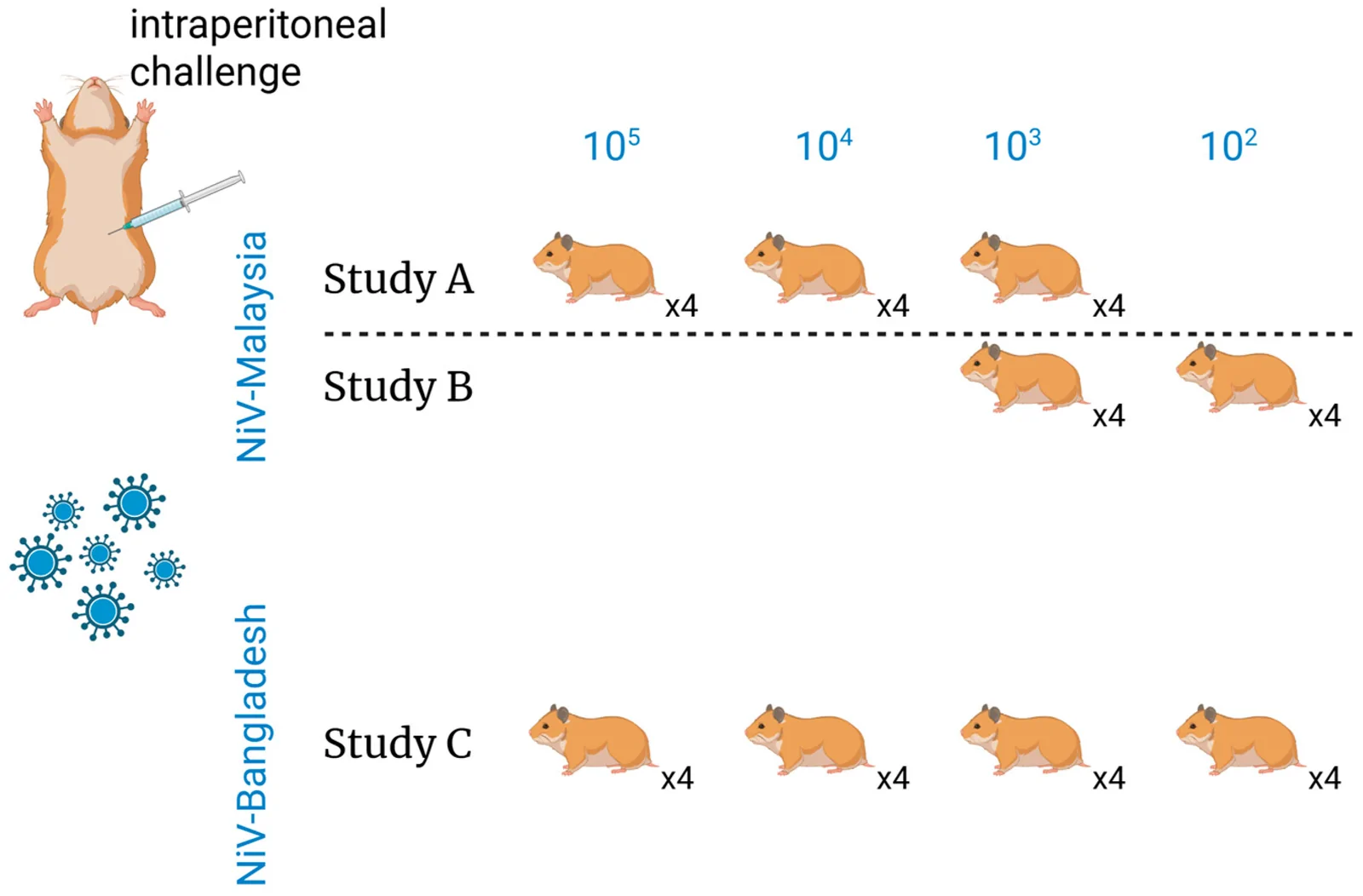
Overview of dose-ranging studies with NiV in hamsters conducted via the intraperitoneal challenge route.

Data are derived from three independent studies (Studies A–C) using a total of 36 hamsters, with challenge doses ranging from 10^5^ to 10^2^ TCID_50_ (Figure 1). The virus was diluted in sterile phosphate buffered saline (PBS; Gibco, Paisley, UK) to obtain the relevant challenge dose. For challenge, 200 µL was injected into the intraperitoneal cavity.

#### 2.4.2. Intranasal Challenge

**Figure 2:**
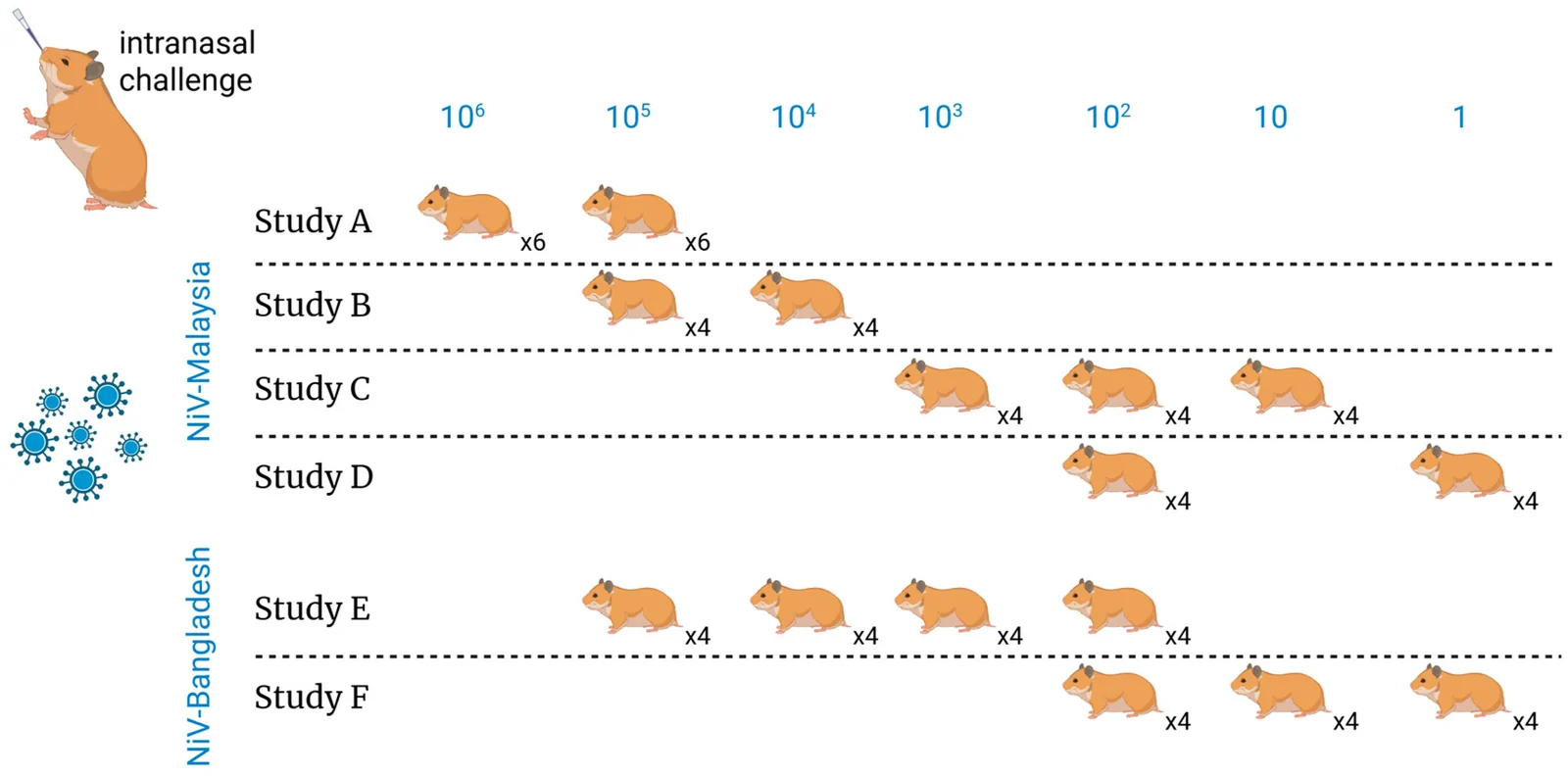
Overview of dose-ranging studies with NiV in hamsters conducted via the intranasal challenge route. NB: Studies D and F were run in parallel and thus count as a single independent study.

Data are derived from five independent studies (Studies A–F) using a total of 68 hamsters, with challenge doses ranging from 10^6^ to 1 TCID_50_ (Figure 2). The virus was diluted in sterile PBS (Gibco, Paisley, UK) to obtain the relevant challenge dose and delivery was performed by instillation in a volume of 100 µL per nostril.

### 2.5. Clinical Observations

At the same time each day (morning) animals were weighed and had temperatures recorded via an implantable ID/temperature chip (idENTICHIP with Bio-Thermal, MSD Animal Health, Milton Keynes, UK). Throughout the studies, clinical signs were recorded at least twice a day by experienced husbandry and animal welfare staff. These scores were converted into numerical values (Table 1), and a cumulative score was then assigned for each animal at that timepoint.

### 2.6. Necropsy Procedures

Upon meeting humane clinical endpoints or at the scheduled end of studies (day 21 post-challenge), hamsters were anaesthetised with isoflurane and then given an overdose of sodium pentobarbital. Tissue samples were collected into histology pots containing 10% neutral-buffered formalin for fixation by immersion prior to histopathological analysis.

### 2.7. Histopathological Evaluation and RNAscope (Viral RNA) Analysis

Tissue samples from the lung, liver, spleen and brain were fixed by immersion in 10% neutral-buffered formalin for a minimum of three weeks, routinely processed into paraffin wax, sectioned at 4 μm, and stained with haematoxylin and eosin (H&E). All slides were digitally scanned using a Hamamatsu S360 digital slide scanner (Hamamatsu Photonics K.K., Shizuoka, Japan) and examined using ndp.view2 software (version 2.8.24; Hamamatsu Photonics K.K.).

In the lung, the severity of broncho-interstitial pneumonia was assessed. Inflammatory infiltrates were evaluated in the liver and spleen, and lymphoid depletion was additionally assessed in the spleen. In the brain, the presence and severity of meningitis and perivascular cuffing were evaluated. Each parameter was scored using a semi-quantitative grading system as follows: 0, within normal limits; 1, minimal; 2, mild; 3, moderate; and 4, marked/severe.

Detection of Nipah virus (NiV) RNA was performed using the RNAscope in situ hybridisation (ISH) technique. Briefly, tissue sections were pre-treated with hydrogen peroxide for 10 min at room temperature, followed by target retrieval for 15 min at 98–101 °C and incubation with Protease Plus for 30 min at 40 °C (Advanced Cell Diagnostics, Newark, CA, USA). Sections were then hybridised with a NiV-specific probe (Cat. No. 439258; Advanced Cell Diagnostics) for 2 h at 40 °C. Signal amplification and detection were carried out according to the manufacturer’s instructions using the RNAscope 2.5 HD Detection Kit–Red (Advanced Cell Diagnostics). Slides were digitally scanned and the presence of viral RNA was analysed using NIS-Ar software v7.0 (Nikon, Praha, Czech Republic), calculating the ISH-positively stained area.

Slide evaluation was conducted by board-certified veterinary pathologists who were blinded to the study groups.

### 2.8. Statistical Analysis

Statistical analyses were performed using Minitab software (version 16.2.2; Minitab Inc., Chicago, IL, USA). Survival data were analysed using Kaplan–Meier estimates with nonparametric distribution analysis incorporating right censoring. Comparisons between groups were conducted using the non-parametric Mann–Whitney U test. A *p*-value ≤ 0.05 was considered statistically significant.

## 3. Results

### 3.1. Clinical Parameters

#### 3.1.1. Intraperitoneal Challenge

**Figure 3:**
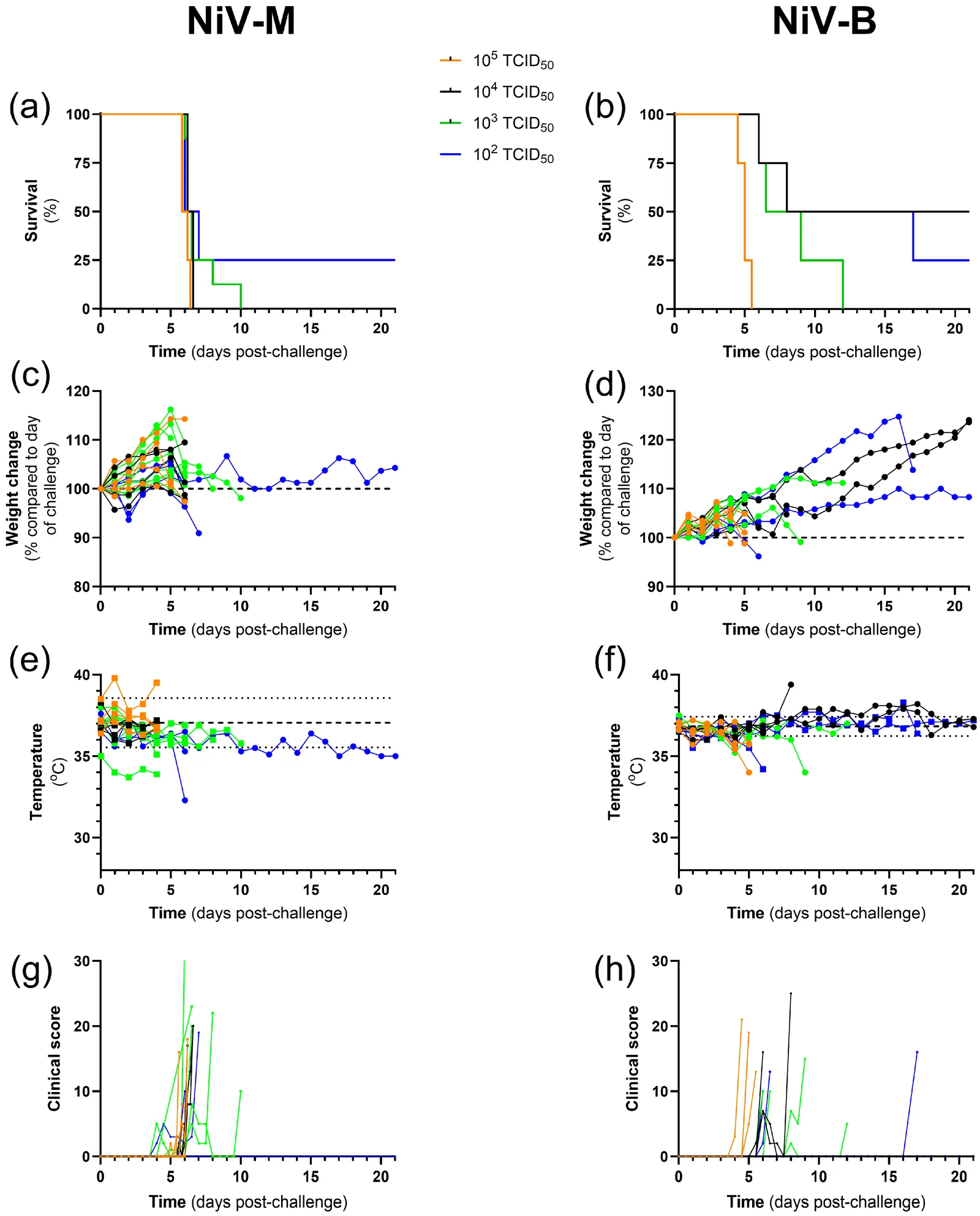
Clinical outcomes after intraperitoneal challenge of hamsters with different concentrations of NiV. (**a**,**b**) Kaplan–Meier survival plots. (**c**,**d**) Change in weight compared to day of challenge. Dashed line represents 100%. (**e**,**f**) Daily temperatures. Dashed line represents mean from all animals on day of challenge with dotted lines showing +/− 2 standard deviations. (**g**,**h**) Clinical scores assessed twice per day. NiV-M and NiV-B refer to the Malaysia and Bangladesh Nipah virus strains, respectively.

Following i.p. challenge, similar numbers of animals reaching humane clinical endpoints were observed between the NiV-M and NiV-B strains (Figure 3a,b). For both strains, a challenge dose of 10^3^ TCID_50_ resulted in uniform severity. For the NiV-B strain, two animals challenged with the higher dose of 10^4^ TCID_50_ survived, indicating variability using this strain and challenge route (Figure 3b). One of these animals showed clinical signs, including those associated with the respiratory system, whereas the other animal remained healthy throughout the study. Animals which met humane endpoints often showed a gradual loss in weight prior to meeting endpoints (Figure 3c,d) or occasionally a rapid drop in body temperature (Figure 3e,f). Clinical signs were of similar level and had a rapid onset (Figure 3g,h).

#### 3.1.2. Intranasal Challenge

**Figure 4:**
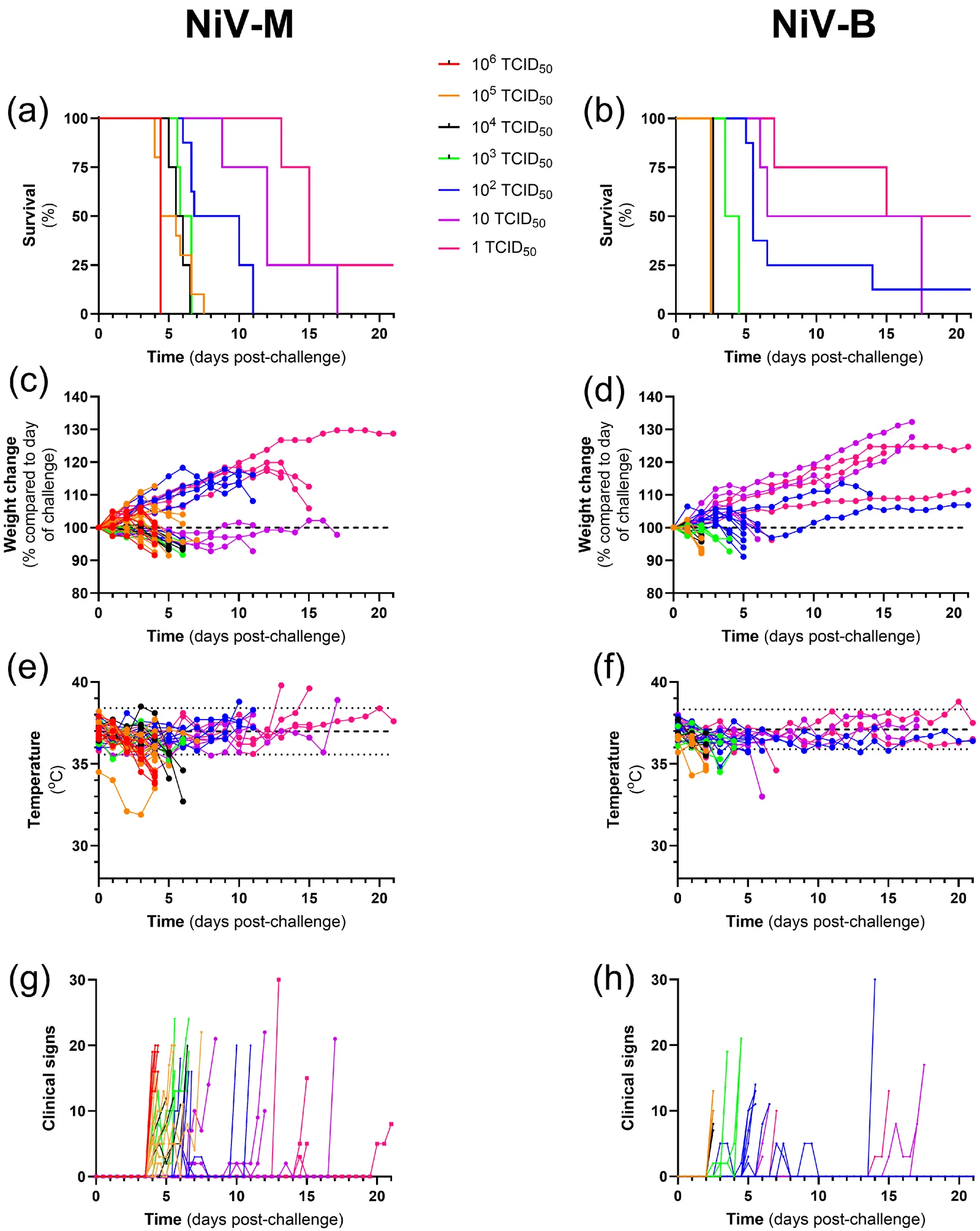
Clinical outcomes after intranasal challenge of hamsters with different concentrations of NiV. (**a**,**b**) Kaplan–Meier survival plots. (**c**,**d**) Change in weight compared to day of challenge. Dashed line represents 100%. (**e**,**f**) Daily temperatures. Dashed line represents mean from all animals on day of challenge with dotted lines showing +/− 2 standard deviations. (**g**,**h**) Clinical scores assessed twice per day. NiV-M and NiV-B refer to the Malaysia and Bangladesh Nipah virus strains, respectively.

Hamsters challenged via the i.n. route with NiV-M or NiV-B at a concentration of 10 TCID_50_ or above all met humane clinical endpoints in a dose-dependent manner (Figure 4a,b). Clinical outputs were similar to i.p.-challenged animals, with observable weight loss in animals (Figure 4c,d), a sudden drop of body temperature on multiple occasions (Figure 4e,f) and a rapid presentation of clinical signs prior to meeting humane endpoint criteria (Figure 4g,h).

### 3.2. Respiratory and Neurological Aspects of Disease

**Figure 5:**
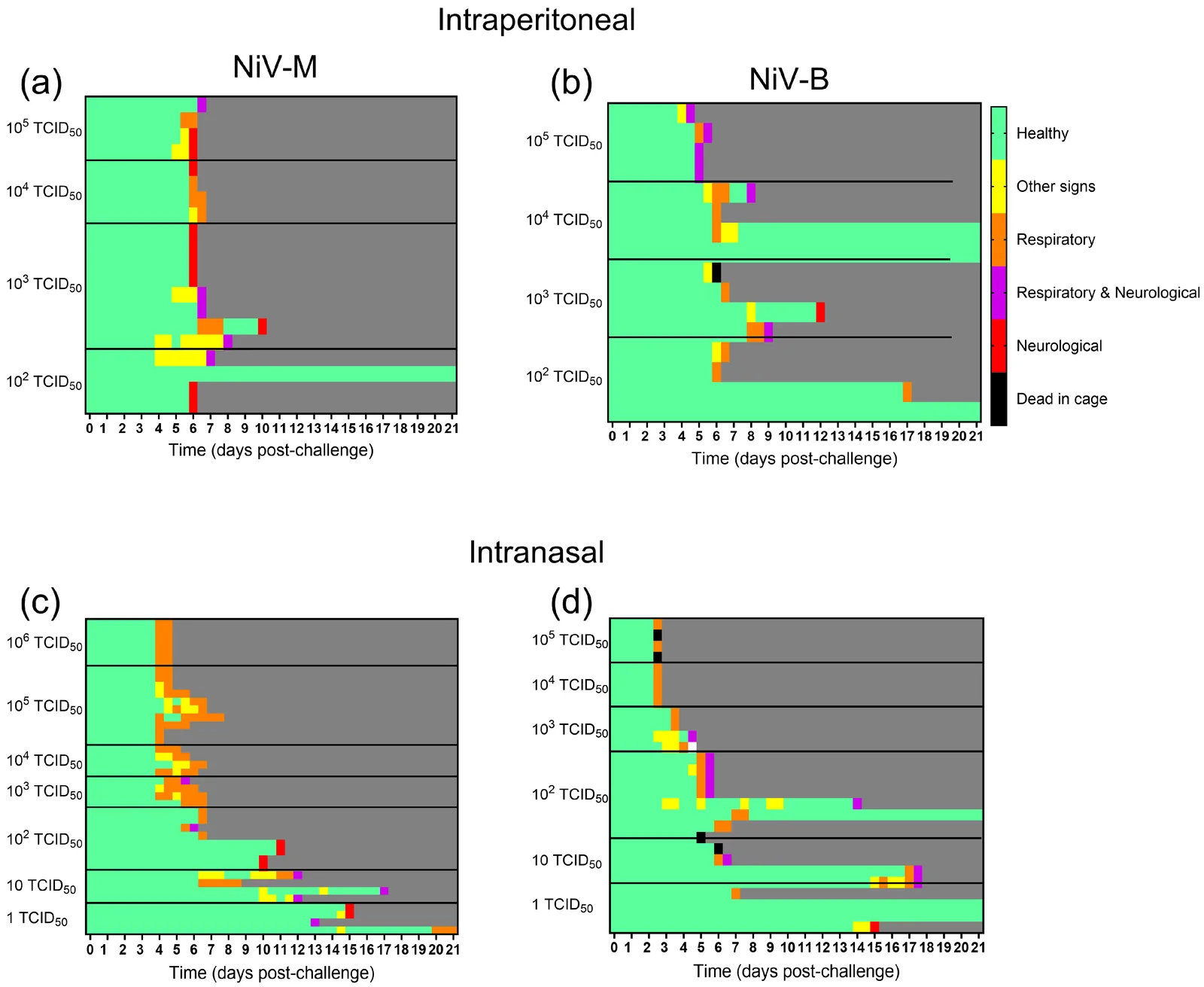
Heatmap plots of clinical signs attributed to respiratory and neurological clinical disease in NiV-challenged hamsters. (**a**) Animals challenged with via the i.p. route with NiV-M and (**b**) NiV-B. (**c**) Animals challenged via the i.n. route with NiV-M and (**d**) NiV-B. NiV-M and NiV-B refer to the Malaysia and Bangladesh Nipah virus strains, respectively.

To ascertain the aspect of disease observed in NiV-challenged hamsters, the clinical signs were grouped into respiratory (wasp-waisted, dyspnoea, rapid breathing) or neurological (ataxia, paralysis, uncoordinated, tremors) categories. For NiV-M, when delivered via the i.p. route, neurological clinical signs were the most evident alongside some respiratory manifestations (Figure 5a). When delivered i.n. at high doses the disease was predominantly respiratory, whereas at lower doses neurological signs were observed (Figure 5c). For NiV-B, both respiratory and neurological signs were recorded after i.p. challenge (Figure 5b). When infected via the i.n. route, similar to findings to NiV-M were observed, where the higher doses resulted in solely respiratory signs whereas for lower doses neurological involvement was increasingly observed (Figure 5d). With NiV-B, five animals were found dead in their cage despite increased welfare monitoring (up to 4 times a day during critical study periods), demonstrating the rapid decline in health of these animals.

### 3.3. Histopathological Changes

**Figure 6:**
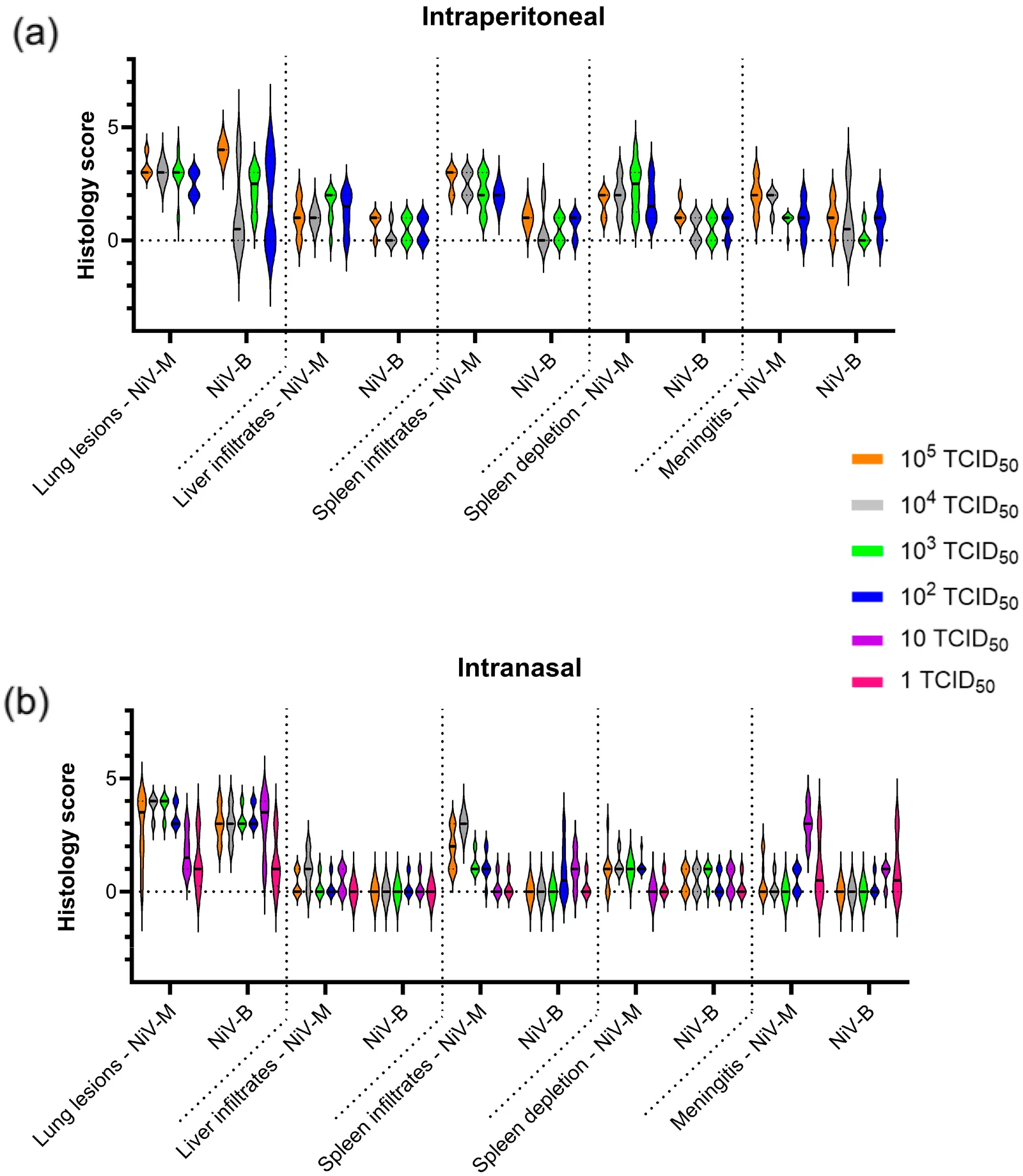
Histology scores from hamsters challenged with Nipah virus. (**a**) Animals challenged via the intraperitoneal route and (**b**) animals challenged intranasally. NiV-M and NiV-B refer to the Malaysia and Bangladesh Nipah virus strains, respectively. Bars show mean values with whisker plots denoting standard error and dots individual results from each animal.

Following i.p. challenge, histopathological changes were evaluated in tissues collected at necropsy (lung, liver, spleen and brain) (Figure 6a). The most consistent and prominent lesions were detected in the lungs, with the high dose (10^5^ TCID_50_) producing moderate-to-severe lesions consisting of broncho-interstitial pneumonia in both NiV-M and NiV-B groups. Liver involvement was comparatively mild, with animals infected with both strains showing mild liver inflammatory infiltrates. Spleen pathology demonstrated distinct patterns for inflammatory cell infiltrates and lymphoid depletion, with NiV-B showing consistently lower infiltrate and depletion scores across doses than equivalent challenge doses with NiV-M. Evidence of central nervous system involvement, assessed as presence of perivascular cuffing and meningitis, was present but generally mild, with NiV-M-challenged animals showing slightly higher scores than those challenged with NiV-B at comparable doses, although variability between individuals was evident.

In contrast, i.n. infection resulted in a more localised disease profile (Figure 6b). Lung lesions were again the dominant feature, with both NiV-M and NiV-B groups showing high histopathology scores at the higher challenge doses. NIV-B demonstrated similarly high lung scores across a broader dose range compared to NiV-M, suggesting efficient induction of pulmonary pathology even at the lower dose of 10 TCID_50_. Systemic pathology following intranasal infection was markedly reduced. Liver infiltrates were minimal across most doses for both strains. Splenic infiltrates were a feature of NiV-M challenge at the higher challenge doses (10^5^–10^4^ TCID_50_), but not with NiV-B, but were then minimal for both strains at lower challenge doses. Splenic depletion was also limited after i.n. exposure, with only mild changes observed at select intermediate doses. Meningitis was infrequently observed following intranasal challenge at higher challenge doses but was detected at lower virus inoculum concentrations with both strains, but highest with the NIV-M group.

**Figure 7:**
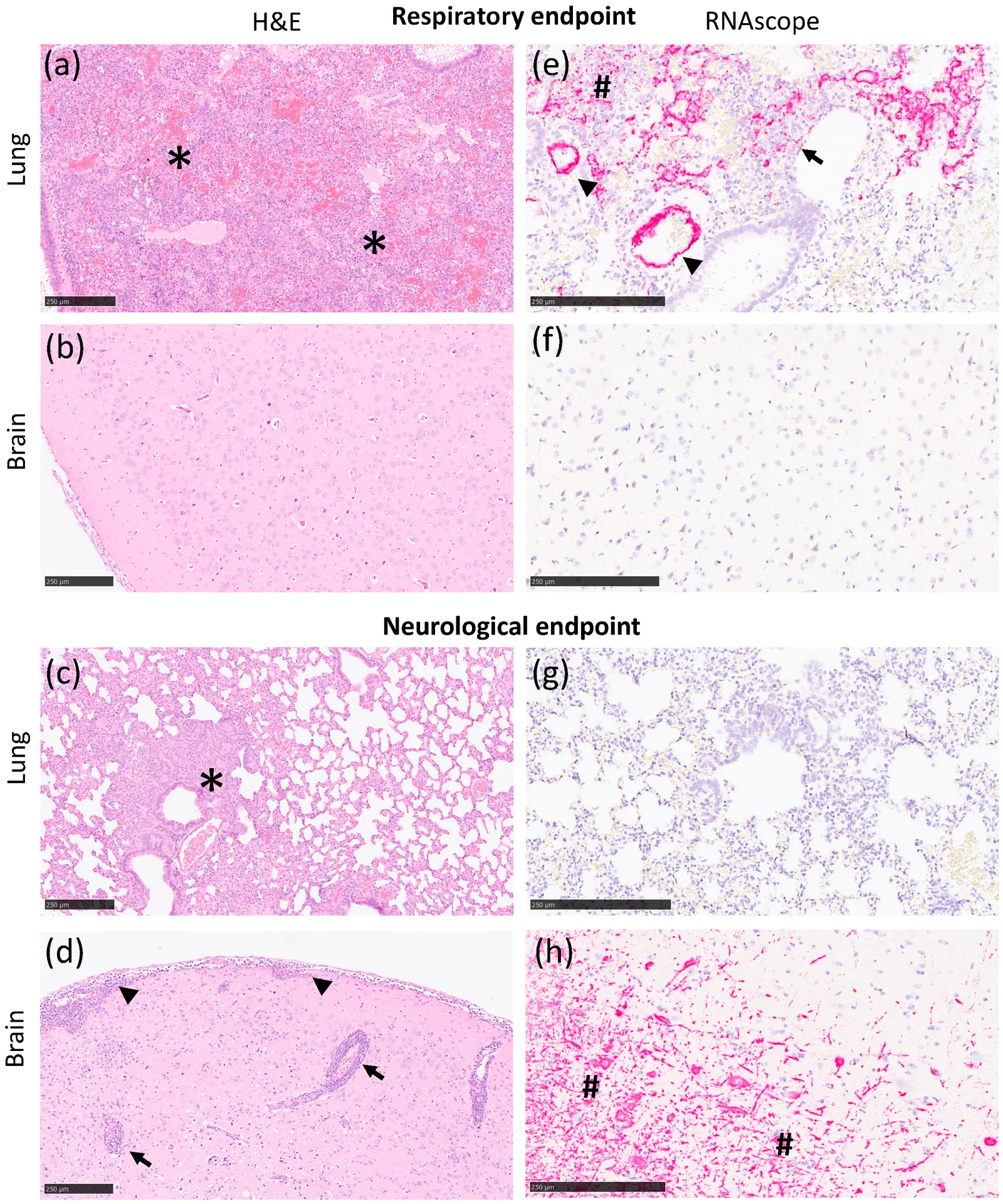
Representative histopathology and RNAscope images from animals which met humane clinical endpoint criteria based on respiratory (**a**,**b**,**e**,**f**) or neurological (**c**,**d**,**g**,**h**) disease signs. (**Left**) H&E staining. (**Right**) RNAscope staining. Animals reaching respiratory endpoints show multifocal/coalescing areas of broncho-interstitial pneumonia ((**a**,**c**), *), with presence of virus RNA in airway epithelia ((**e**), arrows), endothelial cells ((**e**), arrowheads) and inflammatory infiltrates ((**e**), #); however, no nervous system lesion or presence of virus RNA is observed (**b**,**f**). Animals reaching neurological endpoints show mild pulmonary lesions consisting of residual type II pneumocyte hyperplasia ((**c**), *) without presence of virus RNA in the lung (**g**). Neuropathology consist of perivascular cuffing ((**d**), arrows) and meningitis ((**d**), arrowheads), with presence of viral RNA within the endothelial cells, inflammatory infiltrates and neuropil ((**h**), #). Scale bars, 250 µm.

In animals which met humane endpoints due to respiratory disease clinical signs, severe pulmonary histopathological changes (broncho-interstitial pneumonia) were evident (Figure 7a) alongside substantial presence of viral RNA (Figure 7e). The brain showed no significant changes (Figure 7b,f). In contrast, in the animals which were culled due to exhibiting neurological disease manifestations, extensive lesions were observed in the brain (meningitis and perivascular cuffing) (Figure 7d) accompanied by a high burden of viral RNA (Figure 7h), whereas the lungs were only mildly affected (Figure 7c,g).

## 4. Discussion

The present study aimed to define an appropriate challenge dose of NiV in the Syrian golden hamster model, incorporating both i.p. and i.n. routes of infection and two clinically relevant strains, NiV-M and NiV-B. Collectively, the data demonstrate that challenge outcome is influenced by the interplay between dose, route of exposure, and viral strain, so determination of these parameters is essential for generating a reproducible and biologically meaningful disease model suitable for countermeasure evaluation.

A key finding of this study are dose-dependent effects, consistent with previous reports [5,6,8,10,11,12]. Following i.p. challenge, animals consistently met humane clinical endpoints with a dose of 10^3^ TCID_50_, where both strains produced uniform and rapid disease. This aligns with prior reports showing that i.p. exposure results in a consistent lethal outcome and supports the utility of using this route for therapeutic efficacy studies, particularly where robust survival endpoints are required [8]. Unexpectedly, two animals challenged with NiV-B with a higher dose group (10^4^ TCID_50_) survived the full duration of the study. One remained healthy for the duration of the study whereas another exhibited clinical signs, including respiratory, but went on to make a full recovery. Unfortunately, serum was not collected to assess seroconversion in these animals. Due to golden Syrian hamsters not being genetically uniform, animal-to-animal variation may account for these differences in outcome. While i.p. infection reliably produced both respiratory and neurological manifestations in the present study, it represents an artificial route of exposure and may therefore have limited relevance to modelling natural transmission compared with i.n. infection. Due to the mechanics involved with delivery via the i.p. route, there is an increased risk of mis-dosing due to inadvertently introducing inoculum into other compartments (e.g., stomach, intestine) rather than systemically.

In contrast, the i.n. route more closely recapitulates features associated with natural NiV infection, particularly the prominence of respiratory disease at higher challenge doses. Importantly, a clear dose-dependent relationship was observed, with lower doses resulting in increased variability and a shift towards neurological involvement. The viral challenge inoculum resulting in severe disease was lower for i.n. challenge compared to i.p. challenge, with 10 TCID_50_ resulting in all animals meeting humane endpoints with both NiV strains.

The challenge doses which resulted in severe outcomes were considerably lower than those reported by Davies et al., particularly for the i.n. route, where doses of 10^6^–10^7^ TCID_50_ resulted in the majority of animals meeting endpoints, but not 100%, and when 10^2^ TCID_50_ NiV-B was delivered all animals survived [5]. These findings are likely explained by the differences in endpoint criteria. For the project licence of the studies reported within and conducted in the UK, any animals exhibiting neurological clinical signs would be an immediate cull. Others have categorised these neurological manifestations as mild (<30° head tilt, dystaxia, abnormal gait, overgrooming), moderate (30–90° head tilt, circling, ataxia/tremors, paresis, aggressive behaviour, reactive behaviour, absent seizure) or severe (>90° head tilt, paralysis, inability to right self, clonic seizure, unresponsive, unable to obtain food and water) [5]. This reasoning is further supported by another study where euthanasia occurred upon encephalitic signs being observed and demonstrated the same lowest lethal challenge dose of 10^3^ TCID_50_ with NiV-M after i.p. administration and an LD_50_ of 528 TCID_50_ [10], similar to other reports of the LD_50_ being 270 TCID_50_ using the same challenge conditions [12]. Others have reported even lower i.n. and i.p. challenge doses of NiV-M with LD_50_ of <1 and 6 TCID_50_, respectively [13]. For countermeasure evaluation, a model requiring immediate euthanasia upon the onset of any neurological signs will be more stringent than one in which these are categorised and left to further develop. These different endpoints may have an impact on the assessment of interventions due to differing criteria to studies being applied, especially where survival is used as primary output.

The inclusion of both NiV-M and NiV-B strains further underscores the complexity of defining appropriate challenge conditions. Broadly similar dose–response trends were observed between the strains, with both resulting in animals meeting humane clinical endpoints with respiratory and/or neurological clinical signs. This is in contrast to others, where conflicting observations have been reported, with some suggesting a lower pathogenicity of NiV-B compared to NiV-M regardless of challenge route [8,10], whereas in a larger cohort of studies this was supported following i.p. inoculation but was the reverse for i.n. challenge [5].

Histopathological and in situ hybridisation analyses provide further mechanistic insight into the observed disease phenotypes. A more detailed description of histopathological finding in the studies has been previously reported for both the NiV-M [14] and NiV-B [15] strains, so has not been included to prevent duplication. The clear separation between respiratory and neurological disease manifestations in NiV-challenged hamsters—supported by lesion severity and distribution and viral RNA localisation—reinforces the concept of distinct pathogenesis pathways in the hamster model.

From a translational perspective, defining an appropriate challenge dose is critical for ensuring that the hamster model meets regulatory expectations for medical countermeasure evaluation, of which several are being developed [16]. Indeed, the model parameters reported herein have already been used for the assessment of vaccine candidates [17,18]. The data presented here suggest that intermediate challenge doses, which produce consistent but not overwhelmingly rapid disease, may offer the optimal balance for detecting protection and therapeutic benefit.

## Figures and Tables

**Table 1:** Clinical signs and numerical score system.

Clinical Sign	Abbreviation ^1^	Score ^2^
Healthy	H	0
Eyes shut/discoloured	E	1
Ruffled fur	R	2
Lethargic	L	3
Wasp-waisted	W	3
Arched	A	3
Dehydrated	De	3
Unsteady gait (ataxia)	G	3
Ocular	O	3
Temperament change/aggressive	Tp	3
Laboured breathing (dyspnoea)	Lb	5
Rapid breathing	Rb	5
Paralysis	P	8
Uncoordinated	Un	8
Tremors	T	10
Neurological	N	10
Immobile/Death in cage	I, X	10

^1^ Used for recording purposes on clinical record sheets. ^2^ Score values for each clinical sign observed at each timepoint were accumulated to provide an overall semi-quantitative clinical score.

## Data Availability

The data presented in this study are available on reasonable request from the corresponding author.
